# Development and performance of the c4c national clinical trial networks for optimizing pediatric trial facilitation

**DOI:** 10.3389/fped.2023.1302272

**Published:** 2023-12-21

**Authors:** Eva Degraeuwe, Tessa van der Geest, Laura Persijn, Lieve Nuytinck, Ann Raes, Mark Turner, Ricardo M. Fernandes, Johan Vande Walle, Saskia N. de Wildt, IMI2 project conect4children (c4c) consortium, including National Hubs/Networks Belgian Pediatric Clinical Research Network (BPCRN) (Belgium) and Pedmed-NL (Netherlands)

**Affiliations:** ^1^ Faculty of Medicine and Health Sciences, Department of Internal Medicine and Pediatrics, Ghent University, Ghent, Belgium; ^2^ Department of Pediatrics, Ghent University Hospital (UZGent), Ghent, Belgium; ^3^ Department of Pharmacy, Division Pharmacology and Toxicology, Radboud Institute for Health Science, Radboud University Medical Center, Nijmegen, Netherlands; ^4^ Department of Pediatrics and Neonatology, European Rare Kidney Disease Network, Heidelberg, Germany; ^5^ Department of Pediatrics, University of Liverpool (ULIV), Liverpool, United Kingdom; ^6^ Faculty of Medicine, Laboratory of Clinical Pharmacology and Therapeutics, University of Lisbon, Lisbon, Portugal; ^7^ Department of Intensive Care Medicine, Radboud University Medical Center, Nijmegen, Netherlands; ^8^ Department of Neonatal and Pediatric Intensive Care, Division of Pediatric Intensive Care, Erasmus MC-Sophia Children’s Hospital, Rotterdam, Netherlands

**Keywords:** pediatric, drug development, networks, metrics, feasibility

## Abstract

**Introduction:**

The high failure rate of industry-driven pediatric clinical trials leads to insufficient timely labeling of drugs in children and a lack of scientific evidence, resulting in the persistently high off-label drug use. National clinical trial networks can facilitate collaboration between sites, investigators, and experts, increasing the likelihood of successful trials. Within the conect4children (c4c) network, an Innovative Medicines Initiative 2-funded project, National Hubs hosted by National Clinical Trials Networks were set up across 21 European countries to facilitate the setup and execution of pediatric clinical trials. In this paper, we aim to present the performance metrics of the trial feasibility process as well as learnings and challenges encountered by the Belgian and Dutch Networks in working within the European c4c project.

**Method:**

The c4c National Hubs streamline pediatric clinical trials by initiating early country outreach, identifying overlapping studies, recommending quality trial sites, and supporting trial budgeting for both industry and academic settings. To show the impact of Pedmed-NL and Belgian Pediatric Clinical Research Network (BPCRN), internal metrics were collected from 2019 to 2022 on four industry-sponsored and three academic trials performed within the c4c network. Timelines and outcomes of the site identification were collected and analyzed for industry trials. A qualitative analysis was conducted through c4c platforms, sponsor interactions, and stakeholder engagement to evaluate the added value of a research network.

**Results:**

In industry-sponsored trials, full feasibility questionnaires were completed within 2 weeks (*n* = 48), and inclusion rates were up to 80% of clinical sites. Before committing to c4c, 14% of sites were contacted by industry, leading to communication burdens. Utilizing national infrastructure knowledge and therapeutic environment insights helped optimize trial timelines and address feasibility challenges. In addition, national adaptations, such as bilingual staff and site development, played a role in streamlining trial operations in both academic and industry settings. Performance and experiences were similar for both networks.

**Conclusion:**

The early-facilitation examples from the c4c trials demonstrated promising metrics for two National Hubs, including optimized start-up timelines and aiding site selection quality. The learnings and challenges of the Belgian and Dutch Networks provided insights for the development of clinical research networks.

## 1. Introduction

Children represent more than 20% of the population, estimated at 2 billion children globally. However, more than 70% of labeled drugs do not include a pediatric authorization and/or have not been tested in this vulnerable population (1–3.) In Europe, like other continents, substantial efforts have been made to increase the number of pediatric trials and subsequent authorization. The Pediatric Regulations (ECN° 1901/2006) enforced trial sponsors to submit a pediatric investigational plan (PIP) for every novel drug unless a waiver is applied.

In 2017, the European Medicines Agency (EMA) reported that of the 260 new authorized marketing and indications for use by children between 2007 and 2016, a total of 131 PIPs were completed at the end of 2016, meaning pediatric clinical trials from around half of the PIPs remained unexecuted or unsuccessful (4). In 2021, an analysis by Tanemura et al. from the European Clinical Trials Database (EudraCT) suggested that incomplete pediatric trials are primarily associated with the phase of trial design, feasibility, and country eligibility. Low recruitment rate was related to the selection of sites and/or countries with lack of infrastructure and difficult communication (5). These factors led to insufficient scientific evidence for labeling and/or adjusting clinical use of drugs, resulting in a persistently high off-label drug use in children (6, 7).

A critical question remains regarding how to optimize trial conduct. A case study from a Cystic Fibrosis research network (CF Foundation Therapeutics Development) identified effective communication, strong coordination of the trials, and adequate staff as critical success factors (8). However, pediatric clinical trial principal investigators and their site teams are frequently overrun by the burden of a high number of largely different requests for a small number of trial patients, competing with daily clinical work time (9). A need was identified to reduce this burden, by careful planning through homogenizing feasibility processes, staff education, as well as site development for both pediatric-specific sites and sites where pediatrics is a subdiscipline. Support to facilitate communication between sites and sponsors through clinical trial support networks is essential to increase overall efficiency and chances of success.

In the last decade, Europe has experienced the growth of facilitative pediatric clinical trial networks. These networks are initiated across all subdisciplines within pediatrics. A facilitative clinical trial network aims to optimize the design and conduct of clinical trials by providing coordinated and collaborative infrastructure, grouped expertise, and validated sites, which increases the likelihood of successful trials (9–11).

National Networks (NNs) have been founded in several European countries previously and are now working together with newly founded National Networks in a pan-European collaborative network, the conect4children (c4c) consortium (12) (https://conect4children.org/). In the context of the c4c consortium and to improve the clarity of this paper, specific terminology is used when referring to involved networks, which will be designated as “National Hubs” (NH). This term aligns with the standard language used within the c4c initiative when referring to each National Network.

The c4c consortium, founded in 2018, consists of 20 NHs hosted by National Networks across 21 countries and received funding from the Innovative Medicines Initiative 2 (IMI2) Joint Understanding in a private–public partnership (PPP) (Grant Agreement 777389) (12). The consortium assembled expertise and resources from throughout Europe to facilitate and optimize the development and labeling of new treatments for children, primarily through enhancing clinical trial performance and delivery. The project's objective was to develop a sustainable infrastructure that robustly backs multi-discipline pediatric clinical trials, integrate pediatric-specific designs, and reach timely completion. The NHs within the c4c focus on support provided to sponsors and sites during (pre)feasibility questionnaires (FQs), confidentiality disclosure agreement (CDA) process, site acceptance, and contract and budget negotiations. Other trial management activities, such as regulatory submission, monitoring, and daily management of the trial, are the responsibility of the sponsor and not included in the core services of c4c NHs. The Belgian Pediatric Clinical Research Network's (BPCRN) Trial Network upgraded its activity level through this grant support. Also, with upcoming c4c grant support, the Dutch network Pedmed-NL was started in 2017, building on the foundations of the Medicines for Children Research Network, which discontinued after 2011. During the first year, substantial efforts had to be made to rebuild the networks through peer-to-peer personal connection and national roadshows.

Site feasibility is a crucial aspect of trial performance, and one of the first processes explicitly developed and tested in the c4c project. In this paper, we aim to present performance metrics of the trial feasibility process as well as learnings and challenges encountered by the Belgian and Dutch Networks in working within the European c4c project.

## 2. Methods

### 2.1. Trial support by a c4c National Hub

In collaboration with the c4c and within the context of the c4c network, we first describe the trial support activities of an NH using c4c processes. We then present the metrics of the centralized feasibility process of the industry trials, followed by a qualitative analysis of the industry and academic trials, including challenges and learnings of the national hub activities. Activities included in this paper are focused on trial facilitation during the feasibility process as well as analysis of the potential participating sites in Belgium and the Netherlands.

### 2.2. Central organization and site identification facilitation

The process of facilitating academic and industry trials involved a series of evaluations and assessments to ensure the feasibility and eligibility of the trials at each stage of the process. As described by Turner et al., the organization of the c4c trial network is coordinated centrally through a single point of contact (SPoC) and a network infrastructure office (12, 13). The first step was the trial support request, made by the industry sponsor. This request was then evaluated by the Network Management Committee within the c4c, which determined the feasibility and eligibility of the trial based on trial design, patient population, and potential impact on clinical practice (12). To select proof-of-viability (PoV) trials, criteria were designed to evaluate the c4c infrastructure's effectiveness. Both academic and industrial sponsors were required to provide an application with detailed protocols, including endpoints, inclusion/exclusion criteria, timelines, and conduct. In addition to these criteria, the committee also considered different patient age groups, ranging from neonatal to adolescent, and a variety of diseases, from seasonal general to rare ones. This ensured that the c4c infrastructure could handle a broad range of pediatric trials. However, NHs did not participate in incorporating these trials into the c4c network.

**Figure 1:**
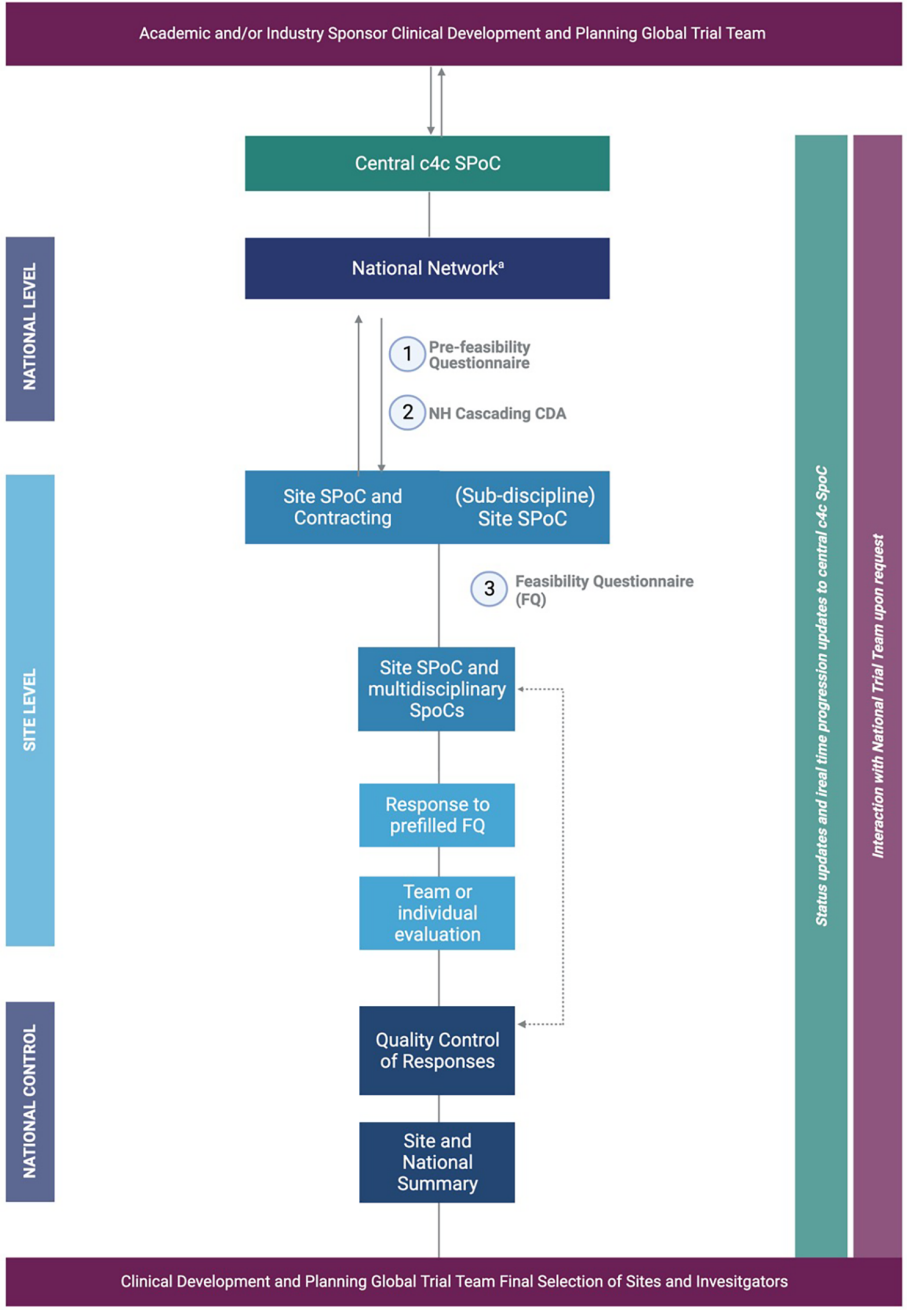
^a^A relevant for the National Network BPCRN and Pedmed-NL. The organization of the c4c trial facilitation through National Networks/Hubs BPCRN and Pedmed-NL. Trial facilitation requests are condensed to the example of a (3) feasibility trial request, which is commenced by a (1) pre-feasibility questionnaire (PFQ) either with the site or on a central and national level as well as a (2) CDA. For clarity, the example of process for facilitation of one site or hospital by one facilitation network is shown. Important is that the central c4c single point of contact supervises every step and the sponsor is involved through either central c4c SPoC and/or global/national teams of the sponsor according to the sponsors wishes and structure. Created by Biorender (2023) from template “flow chart (6 levels, vertical”).

Once a trial was approved to be executed within the c4c network, standardized processes developed within the consortium, including the central feasibility system (CFS) and cascading system shown in Figure 1, were followed. These processes were timeline and metric-driven and focused on performance and quality, including a substation role for the NHs.

### 2.3. Metrics of four PoV industry trials

To assess the impact of NH facilitation during the early phases of country outreach and site identification, we used a selected set of c4c data and outcomes for the c4c industry PoV trials.

Specific metrics data regarding timelines and outcomes of the (pre)feasibility questionnaires, CDA process, site acceptance and contract and budget negotiation, and timelines of trial progression were collected using data from the mandatory timesheets collected within the IMI2 project (as reflected in Table 1) and collated quantitatively (median, minimum, maximum, standard deviation, and 25th percentile, 75th percentile). In the results, the median will be described as mean {±SD, [(25th percentile, 75th percentile)]}. For the inclusion of sites, the number of sites included compared to the total presented sites for all trials were collected in frequency (%). For the inclusion of sites per trial, a division per trial is presented (calculated by the number of selected sites divided by the number of proposed sites, in absolute count).

**Table 1:** Metrics of site identifications for the four PoV trials within the c4c within NH in Belgium and NH in Netherlands.

Activity	NH	Amount of sites	Median (min, max, SD) and [25th percentile, 75th percentile] in calendar days	Comments
CDA between central c4c SPoC and NH In order to discuss confidential information around the topic, to verify the CFS sites, prepare network strategy and review potential hurdles.	BPCRN	—	1 (1, 14, ±6.5) [1, 1]	Early involvement of the network in the trial roll-out and network preparation. An outlier of 14 days was due to learning curve of matching the c4c cascading CDA process with the internal legal processes.
	Pedmed-NL	—	1 (1, 1, N/A) [1, 1]	Early involvement of the network in the trial roll-out and network preparation.
CDA between NH and site In order to share the confidential protocol to each respective trial to complete feasibility questionnaires.	BPCRN	21	1 (1, 4, ±1.2) [2, 12]	Potential delays occurred due to out-of-office of hospital CEOs, which is legally mandatory to complete a CDA per hospital in Belgium and the Netherlands. Other reasons included required additional review of the CDA or educating sites on the cascading process.
	Pedmed-NL	27	1 (1, 9, ±2,69) [5, 26]	Potential delays occurred to departments at sites requiring an additional review of the CDA, aside from the preliminary cascading CDA review.
Feasibility questionnaire Including trial-specific information with regard to recruitment potential, laboratory, and pharmacy requirements, among others. Completion of a feasibility questionnaire requires multidisciplinary information.	BPCRN	21	13 (1, 40, ±9.2) [7, 14]	As mentioned, quality control and summary reports required an additional 2–5 working days. Summer and Winter periods are susceptible to out-of-office periods causing response delays. Pre-filling the questionnaire shortened the timelines.
	Pedmed-NL	27	10 (1, 46, ±9.4) [5.5, 15.75]	Pre-filling the questionnaire shortened the timelines. Performing the quality control and summary reports require an additional 2–5 working days.
Activity	NH	Frequency (%)	Division per trial	Comments
Site selection rates of the completed feasibility questionnaires, the amount of sites that the sponsor had finally included.	BPCRN	67	• Trial 1: 5/6 • Trial 2: 4/7 • Trial 3: 4/6 • Trial 4: 1/2	Reasons for non-inclusion were when sites were selected outside of the c4c procedures (*n* = 3, 14%), meaning sites were included through direct contact and without the c4c facilitation (even though the site is within the NH). Other reasons include capacities of the site (trial start-up, recruitment availabilities, among others). No trial was discontinued.
	Pedmed-NL	80	• Trial 1: 5/5^a^ • Trial 2: 3/5^a^ • Trial 3: 3/4 • Trial 4: 1/1	For trial 1, 13 sites in total completed the FQ but eight signaled that they would not participate with the trial upon protocol revision. For trial 2, an expert network was consulted and a referral model was in place to reduce the necessary sites from 5 to 3. However, after international comparison of the amount of sites the trial was discontinued^a^ for the Netherlands. For the other sites, similar reasons were applicable; however, the vast majority was selected. No sites were included outside of the network.

The comments on the metrics were based on a qualitative analysis of discussions during the National Hub Forum of IMI2 project c4c, the industry PoV consortium meetings that include sponsors direct feedback, and on a national-level discussion with the sponsors’ national representatives. Other comments included the context of the quantitative data with information applicable to both Pedmed-NL and BPCRN. Internal workload analysis, including daily management and quality maintenance of the network, were not included in this analysis. Within Pedmed-NL, two trials were discontinued by the sponsor when compared to the feasibility results in a global setting. The metrics for Pedmed-NL did include the site identification results of these trials sites, as the aim of the metrics is to describe the speed and process of the National Network facilitation, rather than trial-specific complications. Since the sponsor had confirmed which sites they would go forward with, the absolute counts for the division per trial as well as the frequency in % were reported with all five trials included.

## 3. Role of National Hubs

### 3.1. Role of NHs during the four PoV Industry trials

The role of the NHs was key throughout the process of site feasibility and identification. The c4c developed a database of sites and their capabilities (CFS), enabling the preliminary identification of countries and sites based on factors such as patient population, available (human) resources, and local infrastructure. Each NH then had its national expertise, which included site capabilities and resources. At a national level, the NH contributed by reviewing the information collected within the CFS considering the specific trial and suggested essential additions or modifications.

The trial then progressed to a defined site-specific feasibility process, starting with the execution of standardized c4c cascading CDA process that used a CDA template co-developed and approved by the industry sponsors involved in the consortium, NHs, and sites to ensure efficient administration. The central SPoC first signed a CDA with the sponsor. Following this initial step, the central SPoC signed a separate CDA with the NH. Finally, the NH signed a CDA with the individual site potentially participating in the trial. With confidentiality ensured by the fully signed CDA, the study information (e.g., study protocol) could be shared to gain in-depth study knowledge needed to complete the trial-specific questionnaire (TSQ). To fulfill this role, the NH infrastructure was applied to facilitate requests after site identification. Similarly, the multidisciplinary site-level contact points, including subdiscipline contacts, legal departments, pharmacies, and the clinical research coordinators (CRCs), were known contacts to the networks. Knowledge of this national infrastructure was applied in later stages of the trial to support the start-up of the trial and recruitment attainment.

Additional aspects of trial facilitation of the trials within c4c included the following:

- The NHs preferably used a single point of contact at the site level, occasionally complementary to the local subdiscipline and non-pediatric trial supporting departments, such as laboratory and pharmacy.
- The communication and information were tailored according to the country and site requirements in coordination with the sponsor’s central and local trial teams.
- Once the site feasibility responses were collected, the NH teams performed a quality control check, evaluating factors such as completeness, data comparison with other sites and countries (e.g., through consortium or national trial meetings), the logistics, and feasibility of the responses. If needed, the respective single point of contact at the site was contacted for further clarification.
- The BPCRN and Pedmed-NL implemented an additional service to facilitate the completion of the feasibility questionnaire based on previous experiences working in the sites. The NH prefilled the required information as much as possible from an early engagement survey or FQ, so the site staff only needed to validate the information instead of full completion.
- The BPCRN set up a standardized summary report and a process to summarize site and national reports from the feasibility and early engagement process. The process was piloted and optimized by BPCRN and Pedmed-NL and was provided to all c4c NHs to use. The concise summary provided an overview of the advantages, disadvantages, and points of attention per site, as well as recommendations for site selection. This information was beneficial for the sponsor trial teams to make an informed decision regarding non-sponsor selective data. The summary first entailed a national overview of the prevalence and any therapeutic or standard-of-care setting where relevant. Second, the results of the study were presented per site with advantages, disadvantages, and recommendations if a trial is commenced in this site. For example, one site might have had a low experienced principal investigator (PI) but a well-supported system of clinical research nurses and data managers, which should be incorporated in every visit to ensure smooth trial conduct. Third, a conclusion is constructed highly recommended sites and additional inclusions based on the trial’s projections.

Once collected at the national level, the c4c worked centrally to efficiently update and share the information regularly with the global team. This process ensured the quality and completeness of the data collected and facilitated the decision-making process for the global trial team. The evaluation of each trial, whether integrated into the summary or not, was compendiously communicated in standardized calls with the sponsor or the contract research organization (CRO) or provided *ad hoc* upon request. These methods aimed to uphold a transparent and standardized communication protocol and ensured that all relevant parties are informed of trial progression and potential hurdles. Since this process was new for all parties involved, regular meetings and contact with the sites and sponsors were of utmost importance to clarify each role and responsibility, and to prevent doubling in performed tasks, as per the c4c processes. The organization trial service request process through the pan-European system is shown in Figure 1.

### 3.1. Role of NHs during the three PoV academic trials

The feasibility and site identification process for the PoV academWic trials did not follow the CFS system since these trials commenced before the CFS platform was developed. However, the NHs did provide advice regarding potential sites for participation in these trials.

**Figure 2:**
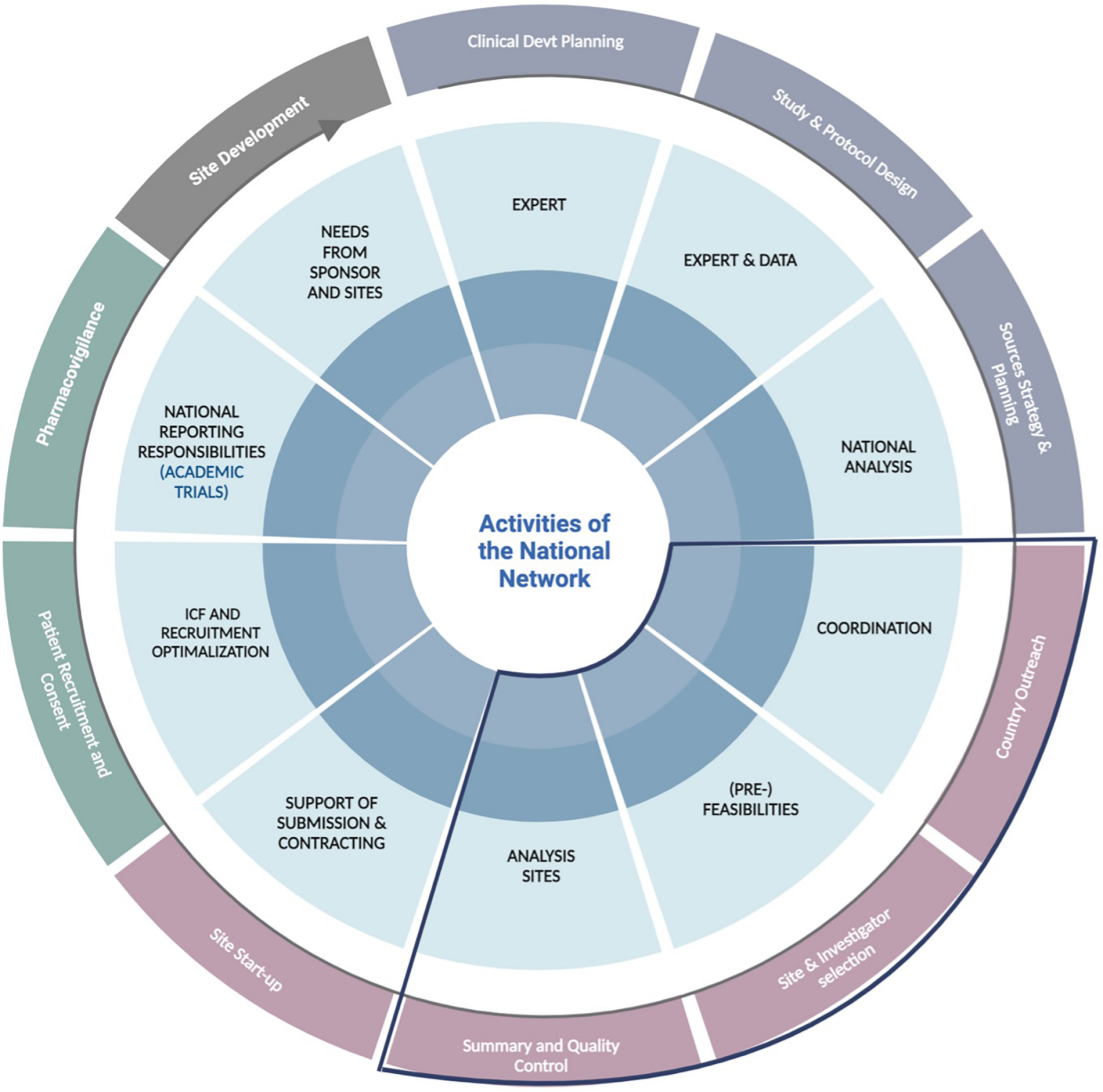
The activities of the National Network situated per phase of the clinical trial process, including support for academic and industry trials. All activities of a National Network are portrayed; yet, the scope of this paper is focused on feasibilities and analysis of sites for (industry) trials (marked in dark blue). Devt, development; ICF, informed consent form.

For the conduct of academic trials, sponsors usually have fewer human resources, lower budgets, and lack of a clinical trial expertise infrastructure to conduct intent-to-label trials. Therefore, the NHs offered customized services for the academic PoV trials, which was more extensive when compared to the services and responsibilities offered to the industry trials. Those facilitative services and responsibilities included (i) site identification and feasibility, (ii) budgeting and contract negotiations with the respective sites, (iii) ethical committee (EC) and national competent authority (NCA) submission, (iv) site start-up visits and preparations, and (v) recruitment planning and troubleshooting. The NHs also contributed to pharmacovigilance activities (14). The total of activities and/or services provided by an NH is shown in Figure 2.

Due to the high variability in the progression of these trials, burdened by COVID-19 and varying sponsor support, a quantitative or metric analysis was not possible. Moreover, the process of standardized timesheets for trial progression was not in place. Timelines considering submission or opening of sites are reported where available. In addition, we will portray a qualitative analysis of where the NHs provided an added value to the academic trials.

## 4. Advantages and learnings of the trial facilitative networks BPCRN and Pedmed-NL

The metrics from the BPCRN and Pedmed-NL were collected for both hubs per industry trial. Internal discussions between BPCRN (ED) and Pedmed-NL (TG) were used to develop the comparison table advantages and learnings for National Networks, including knowledge gained from meetings of the National Hub Forum of the c4c where all 21 NHs were involved.

## 5. Results

### 5.1. Metrics of four PoV industry trials

Metrics of the site identification process for the four PoV industry trials can be found in Table 1. CDA completion had a median of eight calendar days [±8.2, (2, 12)] for BPCRN and a median of 14 calendar days [±18.2, (5, 26)] for Pedmed-NL. CDA signature between NHs and the central SPoC system required less than 6 h. Feasibility questionnaire completion, including a summary report, was completed within a median of 13 calendar days [±9.3, (7, 14)] for BPCRN and a median of 14 calendar days [±9.5, (5.5, 15.75)] for Pedmed-NL]). After analysis of the site identification process for early site identification and/or full feasibility in three clinical trials, NHs comprehensively summarized their findings and presented the sites recommended for trial participation. Of the presented sites, the majority were included in the final site selection, namely, 67% in BPCRN and 80% in Pedmed-NL. However, a total of 14% of sites were contacted separately of the NH during site outreach phase.

### 5.2. Hurdles, timelines, and added value of NHs during the three PoV academic trials

The academic facilitation for the PoV trials performed by both NHs is summarized in Table 2. Hurdles, timelines, and the added value of the NH were portrayed. Aside from COVID-19 disruptions, both NHs were able to progress due to the knowledge of the national infrastructure, therapeutic setting, and knowledge of site's availabilities and resources. BPCRN was the first to receive national approval for the general seasonal trial outside of the sponsor's location (meaning a trial within pediatric common diseases or non-rare conditions, characterized by seasonal fluctuations in occurrence or severity), overcoming the cumbersome investigational medicine product (IMP) issues. Unfeasible studies for a country, e.g., the neonatology trial, caused a clear and early stop in the feasibility process as a competing trial was ongoing in the Netherlands. Moreover, the added value of a national adaptation such as a bilingual staff and necessary site development was essential in optimizing trial timelines.

**Table 2:** Hurdles, timelines, and the added value of the NHs during the academic PoV trials.

Trial type	NH	Hurdles	Timelines	Added value of the NH
General seasonal	BPCRN	COVID-19 disruptions, IMP logistics, privacy adaptations, seasonal disruptions of disease	Approval in 2 months, first country outside of sponsor country; two sites opened by end of 2021	Efficient navigation during pandemic; expedited submission/approval; effective problem-solving
	Pedmed-NL	COVID-19 disruptions, staff shortages, contractual issues, local approval delays	One site opened by July 2022; first recruitment in March 2023	Close relationships with sites and trial support; enhanced trial preparation and coordination; effective budget negotiations; facilitated collaboration
Neonatology	BPCRN	Competing trial, COVID-19 disruptions, transition from paper to digital dossiers	Approval and site opening in 9 months	Bilingual staff; swift submission for neonatal population; knowledge of local trial staff and central coordination
	Pedmed-NL	Competing trial	Not eligible for participation	Early realization of ineligibility due to network knowledge
Rare disease with limited inclusion criteria	BPCRN	Low incidence in sites, upcoming introduction of high-efficacy medication availability	Reapproached in Q1 2021, sites and hub declined in 2 weeks	Quick reassessment of potential sites; understanding of local clinical needs and context
	Pedmed-NL	Sponsor constraints, NCA and EC package sponsor approval delays	Approval in 21 months	Mediation between sponsor and sites; coordination for reimbursement after trial termination

### 5.3. Advantages and learnings of the trial facilitative networks BPCRN and Pedmed-NL

The advantages and learnings identified through the conduct of BPCRN and Pedmed-NL over the last 4 years have been summarized in Table 3. The main advantage was the accessibility to a high-quality and efficient pediatric trial infrastructure, with maintenance and peer-to-peer personal connection. To apply the identified learnings, dissemination of trial facilitation metrics, involvement of multidisciplinary teams (including back-up sites, meaning sites that are qualified to perform the trial but are not considered by the sponsor for opening at the current time), and centralization of trial requests were beneficial.

**Table 3:** Advantages and learnings of NH services from BPCRN and Pedmed-NL.

Topic	Advantages	Learnings	Future perspectives	Stakeholders to overcome pitfalls
Streamlined processes	High quality and speed of service	Multiple checkpoints in verifying delivered data on recruitment and site capabilities ensure the precision and quality in trial estimates based on current and historical knowledge.	Showcasing the improvement/advantages due to NH facilitation (through metrics), which resulted in potential cost reduction and lower trial failure rate for the sponsor.	NH, sponsors
	Continuous communication and information between Central SPoC, NH and site.	More flexible/*ad hoc* capacity within NH needed. Adaptable to the learning curve of collaboration with sponsors.	Standardization of communication process within a NH and multidisciplinary teams. Early inclusion of a NH to connect with the potential sites.	NH, trial sites
Improved feasibility-related data quality	More transparent and reliable data due to standardized process, e.g., CFS.	The NH creates time-efficient and quality completion on site level (Figure 1).	Centralization of all trial requests, from site level as well as from the sponsor level.	NH, sponsor, trial sites
	Potential for having trial-specific peer-to-peer explanation of the trial.	Enhancing knowledge per protocol at NH provides valuable in-depth understanding, and the inclusion of and enchancing the inclusion of sites.	Investment into protocol knowledge was relevant when being consulted for multiple phases of the trial or multiple trials within the same indication.	NH, trial sites
National-specific knowledge	Peer-to-peer personal connection and removal of language barrier.	Importance of maintaining knowledge of personnel on a national-level sustained growth and continuity.	Continuity of the NH team as well as local maintenance of site connection and site engagement.	NH
Site-specific improvement	Support of inexperienced sites	The time-intensive process of naïve site development requires dedicated trial budget	Building a community and interaction between different sites exchanging, including previously naive sites.	NH, trial sites
	Trial-specific advice in terms of trial implementation or recruitment optimization	NHs can advise and link sites to expertise of recruitment optimizations as well as review the sponsors recruitment plan for national adjustments.	Experience of NH broader selection of trials and recruitment analysis, reaching a standardized recruitment process with patient engagement.	NH, trial sites, sponsor
	A continuous and nationally adapted support system that can support the sites with for example budget support.	A NH *ad hoc* approach to site improvement navigation is time-intensive but ensures quality start-up and actual capability of a site.	Allowing a bottom-up approach with regard to engagement to the network and its activities/services. Budget support for both investigator-initiated and industry trials to allow reliable and sufficient budgets.	NH, trial sites
Access to a larger patient pool	By bringing multiple types of institutions together, a larger patient pool can be reached.	Diverse trials, such as those for rare diseases or medical devices, provide unique opportunities to access varied patient pools, needing different type of (quality) sites.	Early involvement of the network in the trial roll-out and network preparation. Progression of more naive sites and/or decentralized infrastructure for long-term follow-up.	Sponsor, NH
Peer-to-peer structure	Access to existing network and trust, improving speed and quality of communication.	Rebuilding connections every 2 years offers the opportunity to refresh relationships and stay updated with evolving needs and trends within the field and the respective network.	Including at least one senior pediatrician within the national hub and involving younger members in the network maintenance.	NN

## 6. Discussion

Incomplete pediatric clinical trials contribute to a substantial gap of empirical evidence, prompting high off-label drug usage in pediatrics (15, 16). The creation of national clinical trial networks has the potential to strengthen collaborations between sites, investigators, and experts, thereby increasing the likelihood of trial success. The Innovative Medicines Initiative 2-funded project, the c4c network, enables pediatric clinical trial delivery through National Hubs that work in 21 European countries (12). The functions of NHs in facilitating trials encompassed early national outreach, site identification, pre-feasibility evaluations, milestone accomplishment, and recruitment strategy enhancements. In this paper, we assessed internal metrics (2019–2022) for four industry and three academic trials supported by Belgium's BPCRN, in comparison with the data from the Netherlands’ Pedmed-NL network. Generally, in terms of metrics and added value, both networks had a similar outcome. Results have been focused on the trial start-up, known as a critical time for the success of a clinical trial (8, 17–19).

In the four PoV industry-sponsored trials, CDAs involving 48 sites across both countries were signed within one to two weeks and full feasibility questionnaires were completed within 1 month. The execution of the CDA cascade process did not align with the intended process across all sites. Certain sites and their legal departments chose to adhere to their own established procedures, resulting in their reluctance to review the cascading c4c template during the initial evaluation or their re-evaluation of previously endorsed agreement templates. Consequently, this non-uniform approach impeded the adherence to the challenging c4c timelines and led to notable standard deviations in the CDA timelines. To mitigate such discrepancies, it is advisable to enhance the preparation of legal departments by the NHs.

Feasibility questionnaires and the preselection of relevant sites by each network led to an inclusion rate of 67% for Belgian sites and 80% for Dutch sites (including discontinued trials) within these trials. However, the inclusion rate in the Netherlands was impacted negatively by a large trial that was halted within the network due to a lack of relevant patients. Furthermore, the utility of site identification summaries, a strategy set up by the BPCRN and piloted by BPCRN and Pedmed-NL, was found to be beneficial for sponsors through direct communication and coherence with potential site selection. Conducting high-quality clinical trial feasibilities and setting expectations, especially in a pediatric setting, has been shown as a key investment to ensure successful trial execution (5, 18, 20). To date, there are no published or publically available metrics to compare a site identification process within a research network setting. However, through internal communication with sponsors, especially within a European setting, these metrics have shown to be very beneficial for fast trial start-up.

Moreover, the necessity of early network consultation from the sponsors and not for “rescue studies” has shown to be imperative for quality facilitation and improving site inclusion rates for the trial. Learning opportunities also emerged for sponsors, whether industry-based or facilitated by CROs, to work synergistically with networks at an early stage and avoid duplicative efforts. An example of duplicating efforts was the industry contacting 14% of sites prior to their commitment to the c4c. Sites that were contacted separately experienced an additional burden, as they had to communicate with three stakeholders instead of one, aside from having to complete a CDA and/or FQ without a fee for every trial request. Moving forward, an introductory call from the network and industry protocols explaining the process of collaboration with sites would be beneficial. It is also vital to identify the stages in the clinical trial process where facilitation by NHs creates the highest gain in efficiency and quality. In summary, early-phase site identification during trial setup and confining site outreach to those within the network have promising metrics to enhance efficiency. These strategies might be encouraging in accelerating trial start-up timelines as well as the potential to improve the quality of site selection. Clinical trials are conducted worldwide, with each continent (and countries) having its own way of organizing and communicating among stakeholders. However, within Europe, a single strategy could potentially be more cost-effective. Industry studies are now managed through the c4c and through existing discipline-specific networks. Sometimes, these studies approach the same centers in Belgium and the Netherlands. To save time, money, and effort, overlapping approaches to the same centers should be minimized.

Despite promising metrics, the NHs had to navigate a steep learning curve. For instance, the clinical trial services within the BPCRN and Pedmed-NL were launched in 2018 alongside the c4c project. The initial metrics should be considered in the context of a learning curve for the network (such as implementing cascading systems, analyzing reports, and managing the IMP), the associated sites, and their PIs. The new approach, where the sponsor communicates through the NH, then to the SPoC, and finally to the PI, was initially treated with natural skepticism by some PIs. Convincing the PIs of the added benefits of NH facilitation required time and persuasion.

### 6.1. Future steps for pediatric clinical trial facilitation

The metrics from our research indicate that the c4c network approach and processes, which support wide-reaching clinical trials and streamlines certain processes like CDA and feasibility checks, have enhanced both speed and quality for the NHs in the Netherlands and Belgium. Conducting the NHs was made possible through substantial in-kind contributions, which should be factored into budget considerations. In upcoming years, the data will be evaluated within other NHs to identify potential areas of improvement in the pediatric setting.

Maintaining the high quality, skills, and expert panel after the c4c grant ends is crucial to ensure the metrics are consistent. The data currently cover c4c viability studies as per IMI regulation, encompassing four dedicated industrial and three academic sponsors. The goal moving forward is to cater to all pediatric studies from foundational European Federation of Pharmaceutical Industries and Associations (EPFA), partners as well as other pharmaceutical companies and CROs across Europe. The benefits of the c4c model, particularly in terms of feasibility and budgeting, need to be communicated effectively. Streamlining all pediatric studies into a single workflow can enhance efficiency, quality, and reduce costs.

One significant hurdle is engaging companies, primarily focused on orphan drugs, which haven't yet been involved but do lack experience in pediatric clinical trials. Conducting trials for orphan drugs presents unique challenges, often necessitating connection to as many potential patients as possible in a country to meet trial requirements. The model proposed by BPCRN and Pedmed-NL emphasizes strong connections to all potential trial sites, ensuring maximum recruitment potential, though this does come with associated costs. It is essential to maintain engagement with these sites. Over the past four years, numerous sites, subdisciplines, and PIs have been approached, but only a select few have seen the rewards for their efforts.

### 6.2. Limitations

This study of facilitative networks has had several limitations. First, the metrics are collected during the development phase of NHs within c4c. All setup processes were new and all involved partners, including industry and sites, had to adapt to that. This led to a steep learning curve, but also in an underestimation of the potential added value of collaboration with the c4c and their NHs, as well as a lack of comparison in terms of other research networks. Relationships between the c4c, sponsors, and hubs were still precarious and needed to grow while further collaborating. Second, the absence of a *post-hoc* analysis after initial site selection restricts the depth of our understanding of site recruitment and evaluation. Third, a comparison to the initial feasibility risk analysis is necessary to validate the feasibility process and input of the NHs. These limitations of our study highlight the need for future adaptability of networks and are currently limited in their ability to predict future circumstances. Future research will need to account for this, incorporating more adaptive strategies, fostering agility, and promoting faster decision-making processes within the start-up networks.

## 7. Conclusion

This study shows promising metrics of two National Hubs based on early-facilitation examples within the proof-of-viability c4c trials. The learnings and challenges of the Belgian and Dutch Networks provide insights for the growth of clinical research networks.

## Data Availability

The raw data supporting the conclusions of this article will be made available by the authors, without undue reservation.
