# Erratum: Development and performance of the c4c national clinical trial networks for optimizing pediatric trial facilitation

**DOI:** 10.3389/fped.2024.1380274

**Published:** 2024-02-12

**Authors:** Frontiers Production Office

**Affiliations:** Frontiers Media SA, Lausanne, Switzerland

**Keywords:** pediatric, drug development, networks, metrics, feasibility

An Erratum on Development and performance of the c4c national clinical trial networks for optimizing pediatric trial facilitation By Degraeuwe E, van der Geest T, Persijn L, Nuytinck L, Raes A, Turner M, Fernandes RM, Vande Walle J, de Wildt SN and IMI2 project conect4children(c4c) consortium, including National Hubs/Networks Belgian Pediatric Clinical Research Network (BPCRN) (Belgium) and Pedmed-L (Netherlands) (2023). Front. Pediatr. 11:1302272. doi: 10.3389/fped.2023.1302272

Due to a production error, the funders “European Union's Horizon 2020 research and innovation programme” and “EFPIA” were erroneously omitted, as well as the disclaimer “This publication reflects the authors’ views and neither IMI nor the European Union, EFPIA, or any Associated Partners are responsible for any use that may be made of the information contained therein”.

The publisher apologizes for this mistake. The original version of this article has been updated.

